# Chronic Lorcaserin Treatment Reverses the Nicotine Withdrawal-Induced Disruptions to Behavior and Maturation in Developing Neurons in the Hippocampus of Rats

**DOI:** 10.3390/ijms22020868

**Published:** 2021-01-16

**Authors:** Magdalena Zaniewska, Agnieszka Nikiforuk, Urszula Głowacka, Sabina Brygider, Julita Wesołowska, Ewa Litwa, Marzena Maćkowiak

**Affiliations:** 1 Maj Institute of Pharmacology, Polish Academy of Sciences, Department of Pharmacology, Laboratory of Pharmacology and Brain Biostructure, 12 Smętna Street, 31-343 Kraków, Poland; urszula.glowacka@uj.edu.pl (U.G.); sabina.brygider@wp.pl (S.B.); mackow@if-pan.krakow.pl (M.M.); 2 Maj Institute of Pharmacology, Polish Academy of Sciences, Department of Behavioral Neuroscience and Drug Development, 12 Smętna Street, 31-343 Kraków, Poland; agnieszka.nikiforuk@gmail.com (A.N.); litwa@if-pan.krakow.pl (E.L.); 3 Maj Institute of Pharmacology, Polish Academy of Sciences, Laboratory of In Vivo and In Vitro Imaging, 12 Smętna Street, 31-343 Kraków, Poland; jwesolow@if-pan.krakow.pl

**Keywords:** 5-HT_2C_ receptor, forced swim test, adult hippocampal neurogenesis, nicotine self-administration, novel object recognition, rat

## Abstract

Preclinical data have shown that treatment with serotonin (5-HT)_2C_ receptor agonists inhibits the behavioral effects of nicotine, including self-administration, reinstatement, and locomotor responses to nicotine. Since the data on the effects of 5-HT_2C_ receptor agonism on nicotine withdrawal signs are limited, we aimed to investigate whether 5-HT_2C_ receptor agonism alleviated the behavioral and neurobiochemical (hippocampal neurogenesis) consequences of nicotine withdrawal in Sprague-Dawley rats. Our data indicate that withdrawal from nicotine self-administration induced locomotor hyperactivity, lengthened immobility time (the forced swim test), induced ‘drug-seeking’ behavior and deficits in cognition-like behavior (the novel object recognition task). A two-week exposure to the 5-HT_2C_ receptor agonist lorcaserin attenuated locomotor hyperactivity and induced recovery from depression-like behavior. Analyses of brain slices from nicotine-withdrawn animals revealed that lorcaserin treatment recovered the reduced number of doublecortin (DCX)-positive cells, but it did not affect the number of *K*_i_-67- or 5-bromo-2’-deoxyuridine (BrdU)-positive cells or the maturation of proliferating neurons in drug-weaned rats. To summarize, we show that lorcaserin alleviated locomotor responses and depression-like state during nicotine withdrawal. We propose that the modulatory effect of lorcaserin on the ‘affective’ aspects of nicotine cessation may be linked to the positive changes caused by the compound in hippocampal neurogenesis during nicotine withdrawal.

## 1. Introduction

Nicotine is the main constituent of tobacco that is responsible for its addictive potential. The development of addiction is closely associated with the rewarding and subjective properties of nicotine, while the possibility of relaxation, decreased appetite and stress symptoms, and mood elevation further strengthen the value of a smoked cigarette [1]. The most common affective withdrawal symptoms that are associated with increased nicotine craving and relapse include stress and negative affect [2,3]. Continuous research focusing on increasing the knowledge about factors that trigger relapse will generate more effective anti-smoking therapies.

Nicotine stimulates nicotinic acetylcholine receptors and leads to an increase in the release of acetylcholine, and indirectly to the release of glutamate, dopamine (DA), and serotonin (5-HT), among other outcomes [3]. The 5-HT system plays a role in the regulation of physiological processes such as sleep, regulation of emotions, learning and memory, and motor behavior. It also plays a role in pathological conditions, including depression, anxiety, and drug addiction [4]. Out of 14 subtypes of 5-HT receptors, the important receptor in relation to the interaction with addictive drugs, including nicotine is attributed to 5-HT_2C_ receptors [5]. The 5-HT_2C_ receptors are densely distributed within the brain regions associated with nicotine effects, and their highest expression is observed in the ventral striatum and the hippocampus.

Preliminary clinical studies have shown that a selective 5-HT_2C_ receptor agonist named lorcaserin (K_i_ = 29 nM; [6]), which in 2012 was approved by the US Food and Drug Administration for treating obesity [5], exhibits positive effects in people addicted to tobacco (such as an increase in smoking abstinence and decrease in associated weight gain) [7,8]. Although more recently the drug was withdrawn from the market due to an increased occurrence of cancer in lorcaserin-treated (10 mg, twice daily) group of patients [9], the effect of a centrally acting agent on the increased incidence of tumors is hard to explain [10].

Preclinical studies demonstrated the inhibitory effect of 5-HT_2C_ receptor agonists (CP-809101, lorcaserin, Ro60-0175, WAY161,503 or WAY163,909) on the following addiction-related effects of nicotine in rodents: nicotine-elicited interoceptive cues, hyperactivity, sensitization, conditioned locomotor activity, impulsivity, intracranial self-stimulation and response to conditioned reinforcement, place preference, self-administration, and reinstatement of ‘drug-seeking’ behavior [11,12,13,14,15,16,17,18,19,20,21,22,23,24,25,26]; many of these behavioral responses were blocked by the selective 5-HT_2C_ receptor antagonist. While most of these analyses were performed after acute drug administration, from the clinical perspective, it seems more important to study the effect of long-term exposure to these compounds (only one study of Levin et al. [13]), which may pose a greater risk of side effects than what is observed following acute treatment. 

Data on the effects of 5-HT_2C_ receptor agonists during nicotine withdrawal are limited. In a model of passive nicotine injections, receptor ligands were shown to reduce the depression-like state during withdrawal at doses that were much lower than those given to drug-naive rats [27], reflecting the enhanced functional response of the 5-HT_2C_ receptor to its agonist during nicotine withdrawal. Also, withdrawal from passive nicotine induced changes in 5-HT_2C_ receptor labeling in the brain, with the strongest change (i.e., decrease in radioligand binding) reported in the hippocampus [28]: this observation suggests that receptor function in the hippocampus may be associated with ‘affective’ aspects of nicotine withdrawal. 

Published data indicate that nicotine self-administration impairs adult hippocampal neurogenesis in rats [29,30,31]. Since abnormal hippocampal neurogenesis seems to contribute to depression [32], it can therefore be assumed that the disturbance in this process that is observed in rats that self-administer nicotine may be linked to a negative state occurring during nicotine withdrawal and that the adjustment of these withdrawal signs could prevent relapse [33]. Concerning the effect of 5-HT_2C_ receptor agonism on hippocampal neurogenesis, there are data showing a decrease in cell proliferation but a simultaneous increase in the maturation of newly generated cells after treatment with WAY161,503 [34], although another study reported no effect of Ro60-0175 on neurogenesis [35]. To date, there are no data on the effects of 5-HT_2C_ receptor agonists on hippocampal neurogenesis in an emotional state during nicotine withdrawal.

Based on the abovementioned considerations, the present study aimed to investigate whether chronic lorcaserin administration modulated affective signs (anxiety-like behavior, locomotor responses, depression-like behavior, nicotine craving) and deficits in cognition-like behavior that occur during withdrawal from nicotine self-administration. To determine the potential neurobiochemical correlates of the effects of lorcaserin on nicotine withdrawal signs (mainly depression-like behavior), the changes in adult hippocampal neurogenesis were studied in rats. 

## 2. Results

### 2.1. Rats Acquired Intravenous Nicotine Self-Administration 

**Figure 1:**
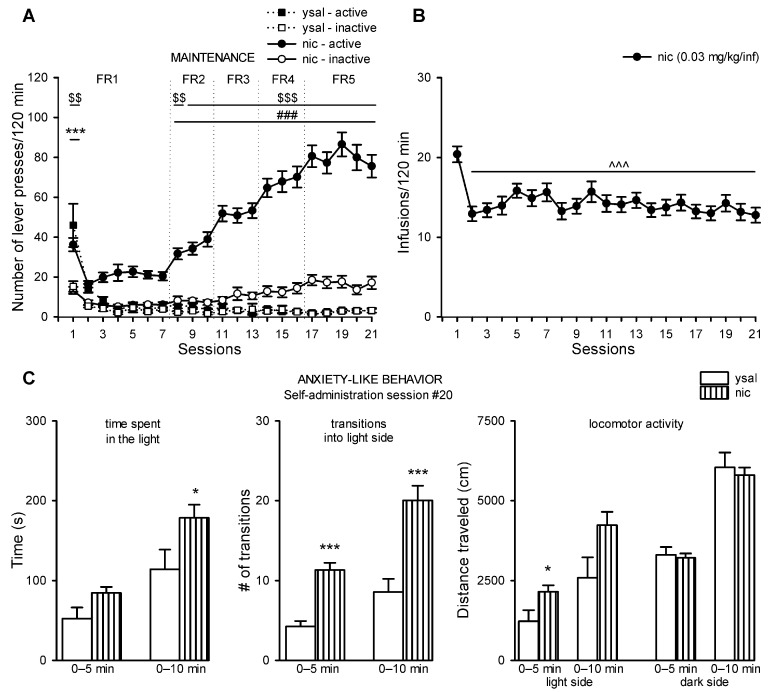
Nicotine self-administration and its effects on anxiety-like behavior in rats. (**A**) The number of lever presses in rats self-administering nicotine (0.03 mg/kg/inf; nic) and rats that received saline (ysal) during an increasing schedule of reinforcement (fixed ratio (FR)1-5). (**B**) Nic intake throughout 21 self-administration sessions. (**C**) Effects of nicotine self-administration on anxiety-like behavior in the light/dark box test (LDB). The data are expressed as the means (±SEM). ysal: *n* = 12; nic: *n* = 30. (**A**) ^***^ *p* < 0.001 versus ysal-inactive; ^$$^ *p* < 0.01, ^$$$^ *p* < 0.001 versus nic-inactive; ^###^ *p* < 0.001 versus ysal-active; (**B**) ^^^^^ *p* < 0.001 versus session 1; (**C**) * *p* < 0.05, *** *p* < 0.001 versus ysal.

Within 21 sessions, nicotine changed the number of lever presses in rats (interaction: F(20,1600) = 15.58, *p* < 0.001). Post hoc Newman–Keuls analysis showed that the number of active lever presses was significantly higher than the number of inactive lever presses in rats that self-administered nicotine (session: 1, 8; *p* < 0.01; sessions: 9–21, *p* < 0.001), and the number was also higher than that of the active lever presses in a ysal group (sessions: 8–21; *p* < 0.001) (Figure 1A). In rats that received passive saline only during session 1, the number of active lever presses was significantly higher than the number of presses on the inactive lever (*p* < 0.001) (Figure 1A). 

The number of nicotine infusions was altered in rats (F(20,580) = 5.47, *p* < 0.001). Post hoc Newman–Keuls analysis showed that the number of infusions during each subsequent session (2–21) was significantly lower than it was during session 1 (*p* < 0.001) (Figure 1B). Total nicotine intake did not differ between the three assigned groups (receiving vehicle or lorcaserin (0.1 or 0.3 mg/kg) during drug cessation). For more statistical details see Supplementary Materials 2.3. and Figure S2. 

### 2.2. Nicotine Exerts Anxiolytic-Like Properties, While its Withdrawal Does Not Induce Anxiety-Like State

Nicotine self-administration during session 20 induced anxiolytic-like properties in the light/dark box test (LDB), as evidenced by: increases in the amount of time spent in the light during a 10 (U = 107, *p* = 0.042)-, but not 5 (*p* > 0.05)-min observation period; number of transitions into the light compartment during a 5 (t = 6.34, df = 38.44, *p* < 0.0001)- and 10 (t = 4.65, df = 35.28, *p* < 0.0001)-min measurement period and locomotor activity in the light side during a 5-min (U = 104, *p* = 0.034), but not during a 10-min (*p* > 0.05) observation period or in the dark compartment (*p* > 0.05) compared to group that received saline infusions (Figure 1C).

**Figure 2:**
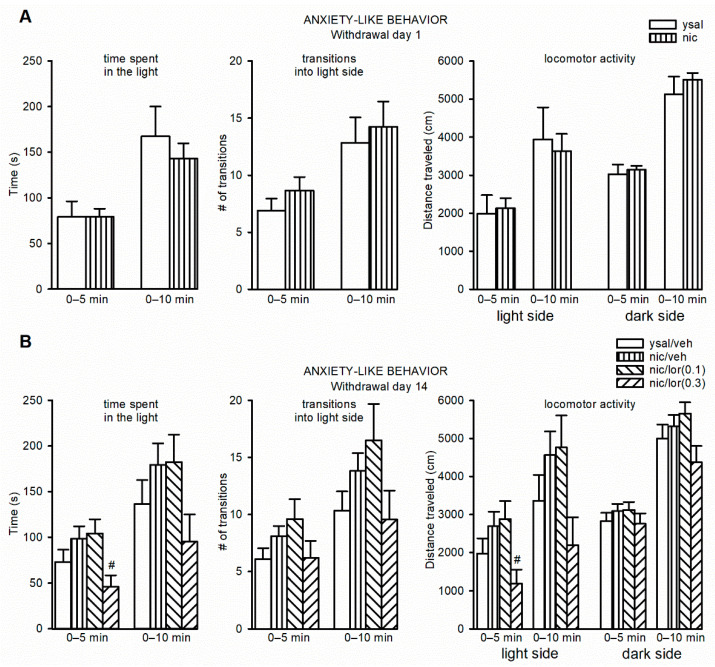
Anxiety-like behavior in the light/dark box test (LDB) in rats withdrawn from nicotine. (**A**) Effects of early withdrawal (day 1) from nicotine (nic) on anxiety-related behavior. (**B**) Effects of chronic exposure to lorcaserin (0.1–0.3 mg/kg, lor(0.1)/(0.3)) on anxiety-like behavior during nicotine withdrawal (day 14). The data are expressed as the means (±SEM). (**A**) ysal: *n* = 12; nic: *n* = 29, (**B**) ysal/veh: *n* = 12; nic/veh: *n* = 11; nic/lor(0.1): *n =* 10; nic/lor(0.3): *n* = 9. (**B**) ^#^ *p* < 0.05 versus nic/veh.

The anxiety-like behavior in rats on day 1 of nicotine withdrawal was not different from the anxiety level in rats that received saline infusions (*p* > 0.05) (Figure 2A). 

During late (day 14) nicotine withdrawal in rats treated chronically (for 13 days) with lorcaserin (0.1–0.3 mg/kg) or a vehicle, the time spent by animals in the light compartment during a 5-min trial was significantly altered (F(3,38) = 3.42, *p* = 0.027). Post hoc Newman–Keuls analysis revealed that animals that were withdrawn from nicotine and were treated chronically with lorcaserin at a dose of 0.3 mg/kg, but not 0.1 mg/kg, exhibited a significant attenuation (*p* = 0.029) in the time spent in the light side compared to that of the nicotine self-administering rats that were given vehicle during withdrawal (Figure 2B). Importantly, the time spent in the light side by rats that self-administered nicotine and received vehicle during drug cessation was not significantly different from that of the ysal group that received vehicle during drug cessation (*p* = 0.20), indicating no changes in anxiety behavior in nicotine self-administering rats on day 14 of drug withdrawal. The time spent in the light compartment during a 10-min observation period did not differ between groups (F(3,38) = 2.11, *p* = 0.11) (Figure 2B). The number of transitions into the light side of the LDB was not changed either in a 5 (F(3,38) = 1.71, *p* = 0.18)- or 10 (F(3,38) = 1.95, *p* = 0.14)-min trial (Figure 2B). The motor behavior of the animals was changed in the light compartment over a 5-min measurement period (F(3,38) = 3.31, *p* = 0.030). The post hoc analysis revealed that administration of lorcaserin at a dose of 0.3 mg/kg, but not 0.1 mg/kg, for 13 days in rats withdrawn from nicotine reduced locomotor activity on day 14 compared to that of the nicotine-deprived rats that received vehicle injections during drug withdrawal (*p* = 0.034) (Figure 2B). The locomotor activity was not altered across a 10-min observation period on the light side of the LDB (F(3,38) = 2.53, *p* = 0.072) or in any trial with the dark compartment (5-min: F(3,38) = 0.67, *p* = 0.57; 10-min: F(3,38) = 2.23, *p* = 0.10) (Figure 2B).

### 2.3. Lorcaserin Reverses the Enhanced Immobility Time during Nicotine Withdrawal

**Figure 3:**
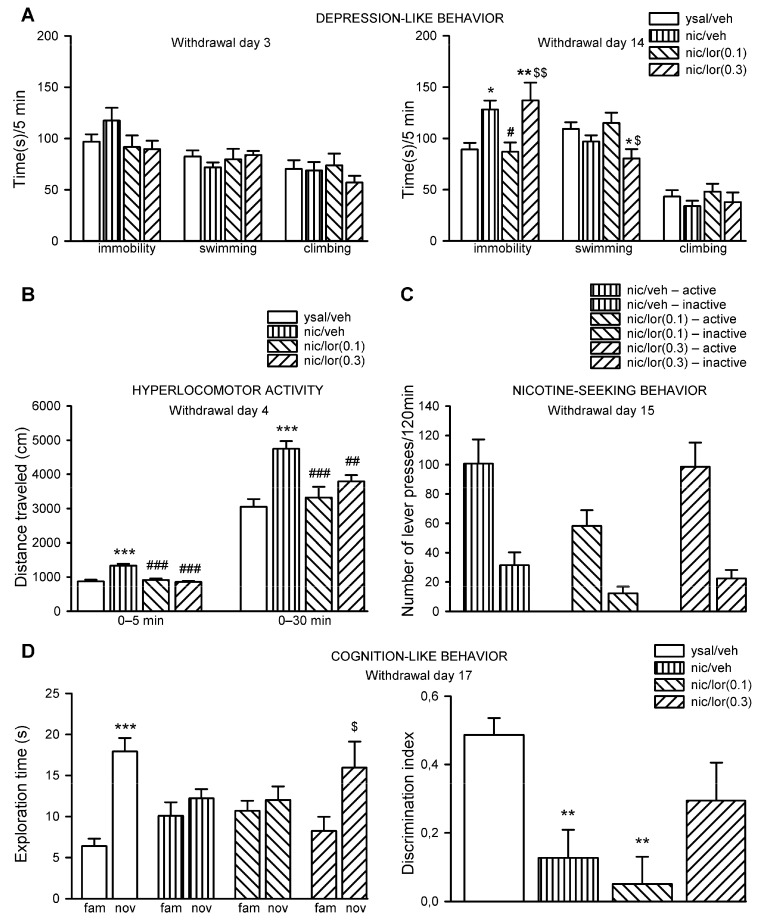
Effects of chronic lorcaserin administration on the behavioral signs of nicotine withdrawal. (**A**) Effects of chronic exposure to lorcaserin (0.1–0.3 mg/kg, lor(0.1)/(0.3)) during nicotine (nic) withdrawal on depression-like behavior in the forced swim test (FST) during early (day 3; left panel) and late (day 14; right panel) drug withdrawal. (**B**) Effects of chronic exposure to lor(0.1)/(0.3) on locomotor hyperactivity during drug withdrawal. (**C**) Effects of chronic exposure to lor(0.1)/(0.3) on lever presses induced by nic priming during abstinence. (**D**) Effects of chronic exposure to lor(0.1)/(0.3) on deficits in cognition-like behavior in the novel object recognition task (NORT) in nicotine-withdrawn rats. The data are expressed as the means (±SEM). (**A**) left panel: ysal/veh: *n* = 12; nic/veh: *n* = 11; nic/lor(0.1): *n* = 10; nic/lor(0.3): *n* = 8. Right panel: ysal/veh: *n* = 12; nic/veh: *n* = 11; nic/lor(0.1): *n* = 9; nic/lor(0.3): *n* = 9. (**B**) ysal/veh: *n* = 12; nic/veh: *n* = 11; nic/lor(0.1): *n* = 10; nic/lor(0.3): *n* = 9. (**C**) ysal/veh: *n* = 12; nic/veh: *n* = 10; nic/lor(0.1): *n* = 9; nic/lor(0.3): *n* = 9. (**D**) ysal/veh: *n* = 12; nic/veh: *n* = 11; nic/lor(0.1): *n* = 10; nic/lor(0.3): *n* = 9. (**A**) * *p* < 0.05, ** *p* < 0.01 versus ysal/veh; ^#^ *p* < 0.05 versus nic/veh; ^$^ *p* < 0.05, ^$$^ *p* < 0.01 versus nic/lor(0.1); (**B**) *** *p* < 0.001 versus ysal/veh; ^##^ *p* < 0.01, ^###^ *p* < 0.001 versus nic/veh; (**C**) Lever effect: *p* < 0.001 active-lever presses versus inactive-lever presses; lorcaserin effect: *p* < 0.05 nic/lor(0.1)–lever presses versus nic/veh or nic/lor(0.3); (**D**) left panel: *** *p* < 0.001 versus ysal/veh–familiar; ^$^ *p* < 0.05 versus nic/lor(0.3)–familiar. Right panel: ** *p* < 0.01 versus ysal/veh.

Analysis of rat behavior during early drug withdrawal (day 3) indicated that there was no change in the behavior of rats in the forced swim test (FST) in all tested parameters, including immobility time (F(3,37) = 1.58, *p* = 0.21), swimming (F(3,37) = 0.62, *p* = 0.60) or climbing (F(3,37) = 0.53, *p* = 0.66) (Figure 3A; left panel).

However, on day 14 of nicotine withdrawal the immobility time was significantly changed in rats (F(3,37) = 5.97, *p* = 0.0020). Post hoc analysis demonstrated that withdrawal from nicotine evoked a significant increase in the immobility time (*p* = 0.013) compared to that observed in rats that received saline infusions (Figure 3A; right panel). Importantly, chronic administration of lorcaserin at a dose of 0.1 mg/kg induced a significant reduction in immobility time (*p* = 0.025) in drug-weaned rats compared to that of self-administering animals that received vehicle during withdrawal (Figure 3A; right panel), indicating an antidepressant-like effect. A higher dose of lorcaserin (0.3 mg/kg) did not change the immobility time of drug-weaned rats compared to that of rats deprived of nicotine that were given vehicle for 14 days during withdrawal (*p* = 0.55); the level of immobility in lorcaserin (0.3 mg/kg)-treated rats was significantly higher than that of control rats that received saline infusions for 21 sessions (*p* = 0.0077) and rats that were treated with 0.1-mg/kg lorcaserin during drug withdrawal (*p* = 0.0098) (Figure 3A; right panel). There was a significant change in swimming in the groups of rats (F(3,37) = 3.55, *p* = 0.024). Post hoc Newman–Keuls analysis demonstrated that lorcaserin at a dose of 0.3 mg/kg significantly reduced swimming in rats compared to what was observed in ysal rats that received vehicle during withdrawal (*p* = 0.034) and the rats of a nicotine group that received a lower dose of the drug (*p* = 0.018) (Figure 3A; right panel). There was no change in climbing in rats (F(3,37) = 0.73, *p* = 0.54) (Figure 3A; right panel). The behavioral phenotype of rats in the FST indicates that 0.1-mg/kg lorcaserin given during nicotine cessation reversed depression-like behavior in drug-weaned rats, while the 0.3-mg/kg dose was inactive in this test. 

### 2.4. Lorcaserin Reduces Locomotor Hyperactivity during Nicotine Withdrawal

Animal’s spontaneous motor activity during a 5-min observation period was significantly altered on day 4 of nicotine withdrawal (F(3,38) = 22.79, *p* < 0.001). Post hoc Newman–Keuls analysis demonstrated that withdrawal from nicotine induced locomotor hyperactivity compared to ysal control (*p* = 0.00012) (Figure 3B). Lorcaserin (0.1–0.3 mg/kg) attenuated the rats’ stimulated motor behavior (*p* = 0.00012 and *p* = 0.00017, respectively) (Figure 3B). Similarly, the locomotion was changed during a 30-min measurement (F(3,38) = 10.02, *p* < 0.0001) (Figure 3B). Post hoc analysis confirmed that the locomotor activity of nicotine-withdrawn rats was significantly enhanced (*p* = 0.00025) compared to that of control rats receiving saline infusions. Administration of lorcaserin (0.1–0.3 mg/kg) reduced the locomotor activity of nicotine-withdrawn rats compared to that of rats receiving a vehicle during withdrawal (*p* = 0.00066 and *p* = 0.0090, respectively) (Figure 3B). Thus, during early nicotine withdrawal, lorcaserin (0.1–0.3 mg/kg) ameliorated the enhanced motor behavior in rats. 

### 2.5. Lorcaserin Nonspecifically Attenuates ‘Drug-Seeking’ Behavior during Abstinence 

Although statistical analysis indicated that lorcaserin treatment did not significantly affect nicotine-paired lever pressing induced by nicotine priming during drug withdrawal (interaction: F(2,50) = 0.88, *p* = 0.42), a significant lever effect was reported (F(1,50) = 44.38, *p* < 0.001), indicating that the number of presses on the active lever in all treatment groups was significantly higher than the number of inactive lever presses (*p* < 0.001) (Figure 3C). Also, there was a significant effect of lorcaserin treatment (F(2,50) = 3.91, *p* = 0.027). Post hoc Newman–Keuls analyses of the main treatment effect showed that chronic lorcaserin (0.1 mg/kg) treatment significantly decreased the number of lever presses compared to that of the control group (*p* = 0.03) or group treated with 0.3-mg/kg dose (*p* = 0.037) (Figure 3C). The observed behavioral changes indicate that 0.1-mg/kg (but not 0.3-mg/kg) lorcaserin reduced active and inactive lever presses, suggesting that it has a nonspecific inhibitory effect on ‘nicotine-seeking’ behavior.

### 2.6. Lorcaserin Partially Attenuates the Cognition-Like Deficits during Nicotine Withdrawal

The exploration time of novel and familiar objects (Trial 2) was significantly changed in a novel object recognition task (NORT) (interaction: F(3,76) = 4.42, *p* = 0.0064). Post hoc analysis showed that control rats, which received saline infusions, spent significantly more time exploring the novel object than they did exploring the familiar object (*p* = 0.00027) (Figure 3D; left panel). In contrast, rats that were withdrawn from nicotine and treated with either a vehicle or 0.1-mg/kg lorcaserin during nicotine withdrawal exhibited an abolished ability to discriminate novel and familiar objects (*p* = 0.81 and *p* = 0.58, respectively) (Figure 3D; left panel). A higher 0.3-mg/kg dose of lorcaserin allowed significant object discrimination (*p* = 0.021) (Figure 3D; left panel), indicating reversal of the disability to differentiate two objects in nicotine-weaned rats. 

The discrimination index (DI), assessed during Trial 2 of the NORT, was found to be changed in nicotine-withdrawn rats (F(3,38) = 6.31, *p* = 0.0014). Post hoc analysis showed that nicotine withdrawal induced deficits in cognition-like behavior, as evidenced by reduced DI (by 73%, *p* = 0.0081) when compared to that of the control rats that received saline infusions (Figure 3D; right panel). Similarly, 0.1-mg/kg lorcaserin evoked a significant reduction in DI (by 90%, *p* = 0.0024) in drug-weaned rats compared to control animals (Figure 3D; right panel). Following treatment with a higher dose of lorcaserin, there was no change in cognitive functions (by 41%, *p* = 0.098) compared to animals that received saline infusions; DI in rats treated with 0.3-mg/kg lorcaserin was not significantly increased (by 123%, *p* = 0.15) over the DI in nicotine-withdrawn animals treated with vehicle during withdrawal (Figure 3D; right panel). Other behavioral parameters assessed in the NORT are described in Supplementary Materials 2.4. and are shown in Figure S3A,B. Overall, the behavioral observations indicate that only 0.3-mg/kg lorcaserin partially attenuated the impairments in cognition-like behavior seen in nicotine-withdrawn rats. 

### 2.7. Lorcaserin Does Not Affect the Proliferation of Newborn Neurons during Nicotine Withdrawal

Animals used in the analysis of hippocampal neurogenesis during nicotine withdrawal self-administered comparable levels of nicotine as did the animals tested in behavioral assays (Figure S4A–C). For more statistical details, see Supplementary Materials 2.5. 

**Figure 4:**
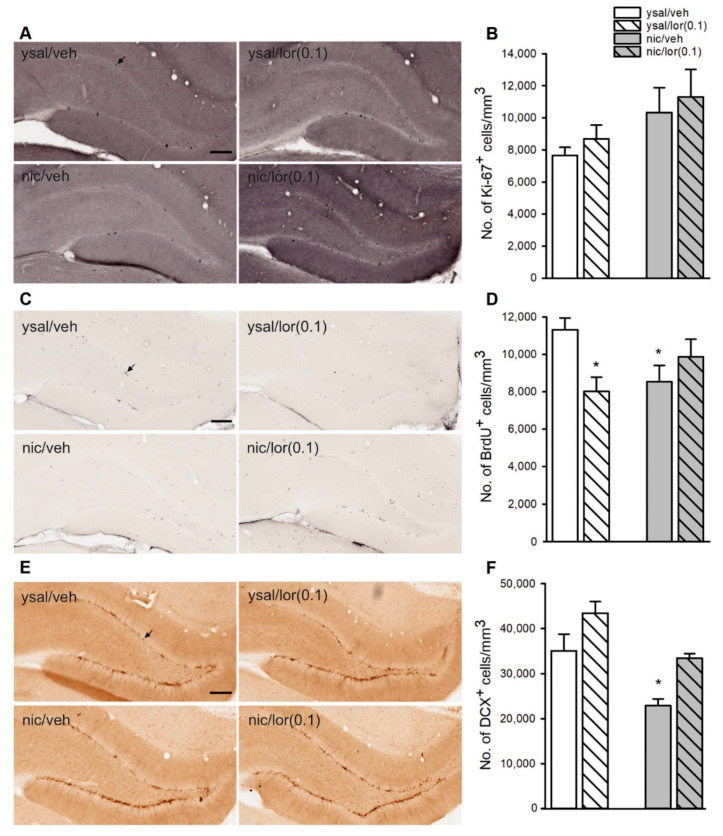
Effects of chronic lorcaserin administration on the number of immature neurons in the hippocampal dentate gyrus (DG) during nicotine withdrawal. (**A**,**C**,**E**) Representative photomicrographs show *K*_i_-67 (a proliferation marker; (**A**)), 5-bromo-2’-deoxyuridine (BrdU, a marker of survival; (**C**)) and doublecortin (DCX, a marker of immature neurons; (**E**)) labeling (indicated by the black arrows). Images were taken with a 20x magnification objective lens. Scale bars: 200 µm. (**B**) Effects of chronic exposure of nicotine (nic)-withdrawn rats to lorcaserin (0.1 mg/kg, lor(0.1)) on the number of *K*_i_-67^+^ cells/mm^3^ in the hippocampal DG on day 14 of nic withdrawal. (**D**) Effects of chronic lor(0.1) on the number of remaining BrdU^+^ cells/mm^3^ in nic-withdrawn rats. (**F**) Effects of chronic lor(0.1) on the number of DCX^+^ cells/mm^3^ during nic cessation. The data are expressed as the means (±SEM). ysal/veh: *n* = 4; ysal/lor(0.1): *n* = 5; nic/veh: *n* = 4; nic/lor(0.1): *n* = 4. (**D**) * *p* < 0.05 versus ysal/veh; (**F**) nicotine withdrawal effect: *p* < 0.001 nic versus ysal; lorcaserin treatment effect: *p* < 0.01 lor(0.1) versus veh; * *p* < 0.05 versus ysal/veh.

Analysis of the brain slices revealed no change in the number of *K*_i_-67^+^ cells/mm^3^ in the dentate gyrus (DG) of the hippocampus of nicotine-withdrawn rats that were treated with lorcaserin during withdrawal (interaction: F(1,13) = 0.0004, *p* = 0.98) (Figure 4A,B), indicating that lorcaserin did not influence the proliferation process during nicotine withdrawal.

### 2.8. Lorcaserin Does Not Alter the Disrupted Survival of Newborn Cells during Nicotine Withdrawal

Although data analysis revealed that chronic lorcaserin affected the number of 5-bromo-2’-deoxyuridine (BrdU)^+^ cells in the DG during nicotine withdrawal (interaction: F(1,13) = 8.07, *p* = 0.014), post hoc Newman–Keuls analysis showed no change in the number of BrdU^+^ cells/mm^3^ in nicotine-withdrawn rats (*p* = 0.075) and the ysal rats that received lorcaserin during drug cessation (*p* = 0.057) when compared to the vehicle-treated ysal group. However, single group comparisons indicated that the number of BrdU^+^ cells/mm^3^ was significantly decreased in nicotine-withdrawn rats (t = 2.57, df = 6, *p* = 0.042) and in ysal rats treated with lorcaserin (t = 3.30, df = 7, *p* = 0.015) compared to that of the ysal rats chronically treated with vehicle (Figure 4C,D). The findings indicate that the reduced survival of newly generated cells in nicotine-withdrawn rats is not altered by chronic lorcaserin treatment. 

### 2.9. Lorcaserin Increases the Number of Immature Neurons in Rats

Although data analysis showed that lorcaserin did not affect the number of doublecortin (DCX)^+^ cells in nicotine-withdrawn rats (interaction: F(1,13) = 0.21, *p* = 0.66), significant effects of nicotine withdrawal (F(1,13) = 20.29, *p* = 0.00059) and lorcaserin treatment (F(1,13) = 14.61, *p* = 0.0021) were reported, implying that both nicotine withdrawal and lorcaserin treatment, independent of the other factor, influenced the number of DCX^+^ cells (Figure 4E,F). As verified by single group comparisons, a significant reduction in the number of DCX^+^ cells was seen in nicotine-withdrawn rats treated with vehicle during withdrawal (t = 3.08, df = 6, *p* = 0.022) compared to that of the vehicle-treated saline group (Figure 4F). In addition, there was no change in the number of DCX^+^ cells in control rats exposed to lorcaserin (t = −1.91, df = 7, *p* = 0.097) (Figure 4F). Thus, nicotine withdrawal decreased and lorcaserin pretreatment increased the number of immature neurons in animals.

### 2.10. Lorcaserin Does Not Alter the Disrupted Maturation of New Neurons during Nicotine Withdrawal

**Figure 5:**
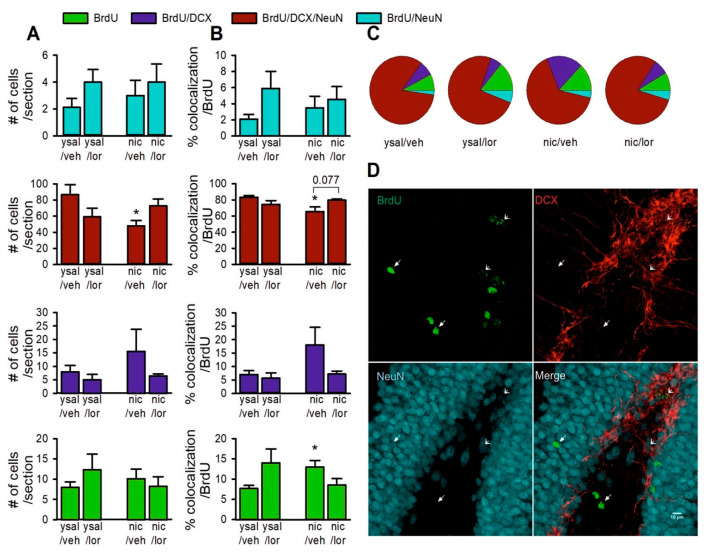
Effects of chronic lorcaserin administration on the maturation of newly generated cells in the hippocampal dentate gyrus (DG) during nicotine withdrawal (day 14). (**A**) Effects of chronic exposure of nicotine (nic)-withdrawn rats to lorcaserin (0.1 mg/kg, lor(0.1)) on the number of proliferating cells (single 5-bromo-2’-deoxyuridine(BrdU)-labeled cells), immature cells (double (BrdU/DCX)- or triple (BrdU/doublecortin(DCX)/NeuN)-labeled cells) or mature neuronal cells (double (BrdU/NeuN)-labeled cells). (**B**) Effects of lor(0.1) treatment on the percent of BrdU, BrdU/DCX and BrdU/DCX/NeuN cell types per total BrdU cell population during nic withdrawal. (**C**) A pie chart showing the percentage distribution of each BrdU cell population. (**D**) Representative confocal microscopy Z-plane stacks of images show BrdU (green), DCX (red) and NeuN (blue) immunofluorescence labeling in the control (ysal/veh) animal (single- or triple-labeled cells indicated by the simple or modified white arrows, respectively). Scale bar: 10 µm. For more details, see Figure 4. The data are expressed as the means (±SEM). ysal/veh: *n* = 4; ysal/lor(0.1): *n* = 5; nic/veh: *n* = 4; nic/lor(0.1): *n* = 4. (**A**) * *p* < 0.05 versus ysal/veh, (**B**) % of colocalized BrdU/BrdU: * *p* < 0.05 versus ysal/veh; % of BrdU/DCX/NeuN colocalization/BrdU: * *p* < 0.05 versus ysal/veh.

Although statistical analysis of the BrdU^+^ cell phenotype in the DG (Figure 5A–D) demonstrated that lorcaserin significantly affected the number of immature neuronal (BrdU^+^/DCX^+^/NeuN^+^) cells/section (interaction: F(1,13) = 6.74, *p* = 0.022), the post hoc test revealed no difference in cell number in nicotine-withdrawn rats compared to the ysal group (*p* = 0.075), and no changes were observed after lorcaserin treatment (*p* > 0.05). As verified by single group comparison, the number of BrdU^+^/DCX^+^/NeuN^+^ cells was significantly reduced following nicotine withdrawal (t = 2.75, df = 6, *p* = 0.033) (Figure 5A). In addition, there was no effect of lorcaserin treatment on the number of immature (BrdU^+^, BrdU^+^/DCX^+^) or mature (BrdU^+^/NeuN^+^) cells/section (*p* > 0.05) (Figure 5A).

Further analysis revealed that lorcaserin administration significantly affected the percent of single-labeled BrdU^+^ cells per total BrdU^+^ cell population in the DG during nicotine withdrawal (interaction: F(1,13) = 5.18, *p* = 0.04) (Figure 5B), though the post hoc analysis did not reveal any significant differences between groups. A single group comparison showed that the percent of colocalization in this population of BrdU^+^ cells was significantly enhanced (by ca. 5%) in nicotine-withdrawn rats (t = −3.04, df = 6, *p* = 0.023) compared to that of the ysal group (Figure 5B,C). There was no effect of lorcaserin treatment on the percent of colocalized BrdU^+^/DCX^+^ cells per total BrdU^+^ cell number (interaction: F(1,13) = 1.98, *p* = 0.18) (Figure 5B). Nicotine withdrawal nonsignificantly increased (by 11%) the percent of colocalized BrdU^+^/DCX^+^ cells/total BrdU^+^ cell number (t = 1.65, df = 3.29, *p* = 0.19) (Figure 5B,C). Lorcaserin administration significantly affected the percent of colocalized BrdU^+^/DCX^+^/NeuN^+^ cells/total BrdU^+^ cell number (interaction: F(1,13) = 7.55, *p* = 0.017) (Figure 5B). Post hoc analysis revealed that nicotine withdrawal significantly reduced (by 18%) the percent of colocalized BrdU^+^/DCX^+^/NeuN^+^ cells/total BrdU^+^ cell number compared to that of the ysal group (*p* = 0.045). There was only an increasing trend (by 14%) in the percent of colocalization in this population of BrdU^+^ cells following lorcaserin exposure in drug-weaned rats compared to that of the vehicle-treated nicotine group (*p* = 0.077) (Figure 5B,C). There was no effect of lorcaserin on the percent of colocalized BrdU^+^/NeuN^+^ cell type per total BrdU^+^ population (interaction: F(1,13) = 0.71, *p* = 0.42) (Figure 5B,C). 

Data analysis demonstrates that the BrdU^+^ cell phenotype, which had been shifted into a more immature state by nicotine withdrawal was not altered by lorcaserin treatment.

## 3. Discussion

Our current data indicate that withdrawal from self-administered nicotine in rats induced locomotor hyperactivity, depression-like behavior, cognition-like deficits, and ‘nicotine-seeking’ behavior, and that a two-week exposure to the 5-HT_2C_ receptor agonist lorcaserin attenuated the locomotor responses and rats’ depression-like state, and, to some extent, the cognition-like deficits. The effects of lorcaserin on nicotine withdrawal-induced neuroadaptations in the hippocampal DG (i.e., the maturation of newly generated neurons) could be related to the drug-induced improvements in the behavioral deficits seen in drug-weaned rats. 

In the current work, animals self-administered ca. 15 infusions per session (ca. 0.45 mg/kg of nicotine/day), which is in agreement with previous observations [30,36]. In contrast to the previous study which showed an anxiogenic effect of self-administered nicotine [36], here we reported the anxiolytic-like state in rats after nicotine infusions (increased time spent in the light compartment, transitions to the light side and locomotor response only in the light compartment of the LDB; present study). The discrepancies between studies may be due to different tests used to evaluate the anxiety-like behavior (LDB; the present study versus social interaction test [36]). 

In accordance with other research groups who previously tested anxiety-like behavior in rats that self-administered levels of nicotine (0.2–0.45 mg/kg/day) comparable to those in our study [36,37], we found that neither early nor late nicotine withdrawal induced alterations in anxiety-like behavior in rats. This is in contrast to the findings of other investigators, which show that withdrawal from nicotine (administered at doses (iv: 1 mg/kg/day; osmotic minipump: 6.5 mg/kg/day) higher than those in our study) induced anxiety-like behavior in rats [37,38]. It thus seems that the appearance of anxiety-related behaviors in nicotine-withdrawn animals depends on the amount of nicotine consumed.

In the current work, we observed that during early (day 4) nicotine withdrawal, rats displayed increased horizontal locomotor activity in a nonfamiliar environment, which is in agreement with previous study [39]; however, there are other reports showing no change or decrease in locomotion [36,40]. Interestingly, we show that the augmented motor reactivity to novelty in drug-weaned animals was attenuated by both doses (0.1 and 0.3 mg/kg) of lorcaserin administered for 4 days. This outcome was expected when considering previous observations that showed inhibitory effects of 5-HT_2C_ receptor agonists on nicotine-related locomotor responses in rats [16,17,21,23]. Such an enhanced locomotor response to novelty is useful for studying sensation seeking in humans [41]. Kahler et al. [42] revealed that increased sensation seeking was a predictor of a reduced smoking cessation rate in humans, while in rats, an increased motor response to novelty contributed to the enhanced social anxiety-like phenotype during nicotine withdrawal [43]. It can therefore be assumed that the inhibitory effect of subchronic lorcaserin on motility in drug-weaned rats reflected a positive rather than negative (i.e., sedative effect) effect on withdrawal signs in animals.

In agreement with previous work [27,44,45], we found that rats withdrawn from nicotine at 2 weeks, but not those in early withdrawal, exhibited exaggerated immobility time in the FST, a behavioral test that is useful for the study of ‘entrapment’ described in depression in humans [46,47]. These findings indicated that nicotine-withdrawn rats developed a depression-like state. In addition, the depression-like phenotype in drug-weaned rats was attenuated by long-term treatment with lorcaserin at a lower dose (0.1 mg/kg), but the same effect was not observed with a higher dose (0.3 mg/kg). Importantly, in drug-naive animals, only a 0.3 mg/kg dose of lorcaserin evoked an antidepressant-like effect, while the highest dose (0.6 mg/kg) induced depression-like behavior, suggesting a narrow therapeutic window for the compound. Our results obtained in drug-weaned rats are in concordance with our previous observations showing that acute treatment with other 5-HT_2C_ receptor agonists (Ro60-0175, WAY163,909) reduced the increased immobility time in rats withdrawn from passive nicotine [27]. The findings support clinical studies that show an improvement in depression symptoms in patients given lorcaserin to treat obesity [48], although one case report showed the development of depressive symptoms after lorcaserin treatment [49]. Thus, our data indicate that chronic lorcaserin administration, only at a subthreshold dose, attenuated the depression-like phenotype observed during nicotine withdrawal. It is noteworthy that the FST design involves memory/learning processes and therefore, drugs that affect memory may lead to false-positive results in this test [50]. Therefore, to avoid misinterpretation of our findings further studies are necessary to verify whether the positive effects of chronic lorcaserin administration in the FST are due to affected memory.

There are several models for studying ‘drug-seeking’ behavior and relapse. ‘Drug-seeking’ behavior can be induced by the drug (e.g., [19,51]), drug-associated cues (e.g., [19]), stress or drug + cue combination [20,24]) either after the withdrawal period in home cages (‘drug-seeking’ during abstinence; e.g., [52]) or after withdrawal combined with extinction phase (reinstatement of ‘drug-seeking’ behavior; e.g., [19]). In the present study, ‘nicotine-seeking’ behavior was induced during abstinence (day 15) by nicotine administration and the immediate introduction of animals to cages associated with previous nicotine infusions. We chose a 0.4-mg/kg dose of nicotine as in our previous studies this dose induced locomotor hyperactivity, behavioral sensitization and served as a discriminative stimulus in rats [15,16,17]. We reported that a two-week exposure to 0.1 mg/kg, but not 0.3 mg/kg, of lorcaserin reduced both active (nicotine-associated)- and inactive-lever presses; the results suggest a low specificity of lorcaserin action on ‘nicotine-seeking’ behavior. Importantly, others who employed extinction phase during nicotine withdrawal have demonstrated that acute administration of 5-HT_2C_ receptor agonists (lorcaserin, Ro60-0175) just before reinstatement of ‘nicotine-seeking’ behavior attenuated active-lever presses, while leaving the inactive-lever presses intact [19,20,24], indicating a specific inhibitory effect of acute treatment with 5-HT_2C_ receptor agonists on ‘nicotine-seeking’ behavior. Importantly, the general reduction in responding (pressing on both levers) following 14-day exposure to 0.1-mg/kg lorcaserin cannot be solely explained by the known inhibitory effect of 5-HT_2C_ receptor agonists on basal locomotor activity [23,24,27], as this drug dose did not alter animals’ locomotor activity measured in the LDB on this day of withdrawal. The lack of selectivity toward the nicotine-paired lever may be also explained by the drug’s inhibitory effect on motor impulsivity [53,54]. In order to objectively assess the role of lorcaserin in this paradigm, further studies on the effects of lower lorcaserin doses on ‘nicotine-seeking’ behavior are necessary.

During protracted nicotine withdrawal, animals exhibited deficits in cognition-like behavior, as evidenced by decreased DI and the inability of drug-weaned rats to distinguish between novel and familiar objects in NORT. These findings are in agreement with those of previous study [44] in which deficits in spatial learning and memory were reported during a similar withdrawal period. Only after administration of a higher lorcaserin dose (0.3 mg/kg) did the impairments in cognition-like behavior in nicotine-withdrawn rats improve, as reflected by the ability to differentiate two objects in these rats. This is in agreement with data in the literature that show a positive effect of 5-HT_2C_ receptor agonist CP809,101 on cognition-like functions in healthy rodents and those with deficits [55,56]. Although data in the literature suggest that cognitive enhancers may prevent nicotine craving and relapse [57], here, we report that the amelioration of the pre-existing cognition-like deficits following 0.3-mg/kg lorcaserin treatment did not influence ‘nicotine-seeking’ behavior. In contrast, the lower lorcaserin dose, which did not alter cognition-like deficits in drug-weaned rats, reduced lever presses induced by nicotine priming during drug cessation. Thus, it can be assumed that the inhibitory effect of lorcaserin on ‘nicotine-seeking’ behavior was not due to its positive effect on cognitive functions. 

It seems important to point out two distinct responses of 0.1- and 0.3-mg/kg lorcaserin in behavioral tests—i.e., the lack of a dose-response effect after chronic lorcaserin administration seen in the present study—which is in contrast to the previously demonstrated dose-related effects of acute lorcaserin [11,13,20,24,25] albeit not in every behavioral paradigm [12]. Considering the fact that lorcaserin also binds to 5-HT_2A_ (K_i_ = 159 nM) and 5-HT_2B_ receptors (K_i_ = 190 nM) (a > 5-fold lower affinity than to 5-HT_2C_ receptors; [6]) and that chronic 5-HT_2C_ receptor agonist administration leads to receptor downregulation [58], we may consider that the shift in the behavioral effects at a higher dose (0.3 mg/kg) of lorcaserin in the FST and ‘nicotine-seeking’ model (loss of the drug’s effect) or NORT (revealing the drug’s effect), following its chronic administration, may suggest the action of lorcaserin at other non-5-HT_2C_ receptors [55]. Another explanation for the diverse effects of lorcaserin may be that, depending on the drug dose, different brain areas can be activated [59]. 

From a therapeutic point of view, greater efficacy is achieved with a lower (0.1-mg/kg) dose of lorcaserin, as it eliminated locomotor responses and depression-like state in nicotine-withdrawn rats. Considering the potential underlying mechanism of action of lorcaserin, different brain regions must be taken into account. Concerning the locomotor hyperactivity which is thought to reflect enhanced DA neurotransmission in the nucleus accumbens [60], indeed, there are supporting data that show that the increase in animals’ motor behavior during early nicotine cessation may be related to dysregulation in the striatal DA system [39]. Recently, it has been shown that acute treatment with lorcaserin attenuated the activity of DA neurons in the ventral tegmental area (brain region rich in 5-HT_2C_ receptors localized on tegmental GABA interneurons, their terminals, and on DA cells [61,62]), but did not affect DA release in the striatum [63]. Future studies will verify whether the drug reduced the enhanced locomotor activity in nicotine-weaned animals by influencing DA neurotransmission in the striatum.

Considering the neurobiochemical correlates of the positive effect of lorcaserin on depression-like behavior during late nicotine withdrawal, we focused in the current study on neurogenesis in the hippocampal DG, which is a region of the brain connected to emotions. We reported that on the same day of nicotine cessation as in behavioral studies, the baseline number of proliferating cells (*K*_i_-67^+^) was not disturbed, whereas the number of surviving (BrdU^+^) and newly generated immature/migrating (DCX^+^) neurons was decreased in nicotine-withdrawn rats. In addition, nicotine withdrawal shifted the phenotype of the remaining BrdU cells into a more immature state (i.e., more BrdU^+^ and BrdU^+^/DCX^+^ and less BrdU^+^/DCX^+^/NeuN^+^ cell types). These findings suggest that withdrawal from nicotine led to a decline in the survival and maturation of new neurons in the hippocampus but did not alter the proliferation of precursor cells. Data in the literature have shown that nicotine self-administration, depending on the experimental setup, attenuated (limited access to nicotine: ca. 0.2–0.6 mg/kg/day; [29,31]) or enhanced (extended access to nicotine: 0.9 mg/kg/day; [30]) different stages of adult hippocampal neurogenesis. It could therefore be postulated that in the current work, decreases in the maturation of newly generated neurons in the DG may arise from nicotine self-administration per se, but not from drug withdrawal. However, our detailed analysis of hippocampal neurogenesis at different time points of nicotine cessation revealed that such an outcome is a protracted withdrawal-induced phenomenon (Zaniewska et al., personal communication). 

To our knowledge, this is the first study showing that chronic administration of lorcaserin in rats withdrawn from nicotine reversed the reduction in the number of immature cells (DCX^+^ cells), leaving cell proliferation and survival in the hippocampal DG intact. It must be added that there was a trend in shifting the BrdU^+^ cell phenotype into a more mature type (increased colocalization of BrdU/DCX/NeuN cell types) after chronic lorcaserin administration. Thus, it can be assumed that the main target of the compound are immature neurons expressing DCX. The positive influence of lorcaserin on the maturation stage of developing cells is partially in line with previous data that showed a shift of the phenotype of mouse neuronal cells toward a more mature type after acute treatment with a 5-HT_2C_ receptor agonist WAY161,503; however, after 7 days of administration of this compound or after acute or chronic administration of a less selective 5-HT_2C_ receptor agonist Ro60-0175 in rats, no effect was observed [34,35]. The inconsistencies between the studies could arise from species differences, drug selectivity or exposure time as well as from the analyzed population of proliferating cells (dorsal versus entire DG). 

Since abnormal hippocampal neurogenesis appears to be linked to depression [32], it can be assumed that abnormalities in this process observed in rats withdrawn from nicotine may be associated with the animals’ emotional state during this period. One of the neurotransmitter systems that is correlated with depression in humans [4] and depression-like behavior in laboratory animals [64] may be the 5-HT system. In a rat model useful for the study of depression, the antidepressant drug fluoxetine, which potentiates 5-HT neurotransmission (selective 5-HT reuptake inhibitor; SSRI), corrected the decreased 5-HT levels in the hippocampus and alleviated depression-like symptoms [64]. Additionally, during nicotine withdrawal, administration of another SSRI, paroxetine, restored depression-like symptoms and reduced 5-HT content in the diencephalon [45]. Although there is some evidence for decreased 5-HT levels in the hippocampus during early nicotine withdrawal [28,65], to date, the levels of neurotransmitters in the hippocampus during protracted withdrawal are not known and remain to be established. With regard to the consequences of reduced 5-HT levels on hippocampal neurogenesis, there are conflicting results: a decline, no effect and an increase in neurogenesis have been reported, but these discrepancies may be partially explained by compensatory changes that occur in genetic models due to life-long 5-HT depletion. In turn, other studies clearly indicated that fluoxetine potentiated hippocampal neurogenesis; similar to the therapeutic effect, this observation occurred after prolonged drug treatment [66].

The abovementioned considerations suggest that the potential new mechanism by which lorcaserin reduced depression-like behavior during nicotine withdrawal may be that reduced 5-HT levels in the hippocampus are replaced by the 5-HT_2C_ receptor agonist lorcaserin. The 5-HT_2C_ receptors are predominantly distributed on adult granule cells of the hippocampal DG [34]. It can therefore be hypothesized that activation of these receptors by lorcaserin, presumably through a direct effect on neighboring differentiating immature cells expressing DCX ([34], present study), could exert a positive influence on hippocampal neurogenesis. Studies with the use of inhibitors of the adult hippocampal neurogenesis are necessary to correlate the neuroadaptations in the hippocampus with drug-induced behavioral outcomes. 

Importantly, loss of newly generated cells in the hippocampus has been shown to be crucial for vulnerability to relapse, and the reversal of these changes may decrease the relapse risk [33]. Thus, it can be postulated that impairments in hippocampal neurogenesis during nicotine withdrawal could be a contributing factor that triggers nicotine craving and relapse. However, to support this hypothesis much work is required. 

In summary, we showed in this study that rats withdrawn from nicotine self-administration exhibited enhanced locomotor reactivity to a novel environment, depression-like behavior, ‘nicotine-seeking’ behavior and impairment in cognition-like behavior. Our data revealed that chronic treatment with the 5-HT_2C_ receptor agonist lorcaserin ameliorated nicotine withdrawal-induced locomotor hyperactivity, depression-like behavior and, partially, cognitive performance. We further suggest that the positive effect of lorcaserin on changes to hippocampal neurogenesis during nicotine withdrawal may be linked to the modulatory effect of the receptor agonist on the ‘affective’ aspects of nicotine abstinence. 

Certain limitations of the present study must be taken into consideration. Access to nicotine was limited to 2 h daily for a total of 21 sessions. Also, to evaluate withdrawal signs in rats, the screening tests, such as the FST or NORT, were applied. We believe that the findings of the present study will provide a promising base for future work, using extended-access nicotine self-administration in combination with tests useful for the study of other symptoms of depression (e.g., the sucrose preference test) and cognition-like deficits (e.g., the Morris water maze test). 

When considering the use of lorcaserin for smoking cessation therapy, one should consider its narrow therapeutic window and potential carcinogenicity [9]. Taking into account the carcinogenicity studies in rats showing that lorcaserin at clinical (10 mg/kg) or higher doses was associated with an increased incidence of cancer [67], the risk of tumor following administration of much lower 0.1-mg/kg dose of lorcaserin as in the present study seems to be rather low. Nevertheless, analysis of the effect of chronic 0.1-mg/kg lorcaserin administration on cancer cell lines will help to determine whether this drug dose is within a reasonable safety margin.

## 4. Materials and Methods 

### 4.1. Animals

Experiments were conducted on adult male Sprague-Dawley rats (*n* = 59; 201–290 g) acquired from a licensed animal breeder (Charles River, Sulzfeld, Germany). A total of 3–4 animals were housed per cage (before the surgery), or they were housed individually (after the surgery) in a colony room (temperature: 22 ± 2 °C; humidity: 55 ± 10%; 12-h light–dark cycle; lights on at 6:00 h). The animal’s condition was monitored once or twice (after the surgery) daily by the experimenter. Rodent chow and water were available ad libitum except for during the initial training sessions (Section 4.3.1). All experiments were conducted during the light phase of the light–dark cycle. The experiments were performed according to the EU Directive 2010/63 EU for animal experiments and were approved by the II Local Ethics Commission at Maj Institute of Pharmacology, Polish Academy of Sciences (permission numbers: 118/2018, approval date: 15 March 2018; 252/2018, approval date: 09.08.2018). All animal studies comply with the ARRIVE guidelines. All the efforts were made to minimize suffering and the number of animals used. The humane endpoints, such as post-operative sepsis, rapid weight loss of 20% of the body weight within a few days, and symptoms of pain were the basis for earlier termination of the procedure. 

### 4.2. Drugs

The following drugs were used: a thymidine analog that labels proliferating cells-(BrdU; Sigma-Aldrich, USA), (-)-nicotine ditartrate (Tocris Bioscience, Bristol, UK, cat no. 3546) and lorcaserin hydrochloride (MedChemExpress; Monmouth Junction, NJ, USA). For more details of drug preparation, the doses of nicotine (expressed as the free base) and lorcaserin, see Supplementary Materials 1.1. and previous publications [11,12,20,24,27,30,68].

### 4.3. Behavioral Signs of Nicotine Withdrawal

#### 4.3.1. Training and Self-Administration 

**Figure 6:**
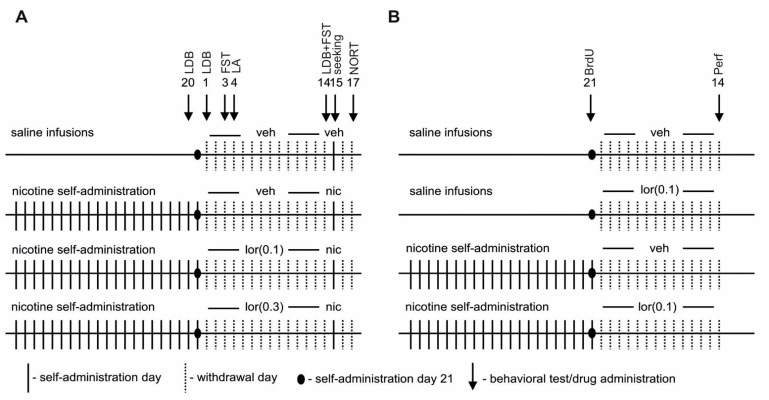
Experimental schedule of nicotine self-administration and behavioral and neurobiochemical analyses performed during nicotine withdrawal. Rats were allowed to self-administer nicotine (0.03 mg/kg/inf, nic) or they received saline infusions (ysal) in 2-h sessions. After 21 sessions, rats entered the withdrawal phase (for 14 days), during which they received daily injections of lorcaserin (0.1–0.3 mg/kg, sc, lor) or a vehicle (veh). (**A**) Effects of lor (0.1–0.3 mg/kg) on the behavioral symptoms of nic withdrawal. The following symptoms of nic withdrawal were measured: anxiety-related behavior in the light/dark box (LDB, the 20th self-administration session; withdrawal day 1 and 14), depression-like behavior in the forced swim test (FST, withdrawal day 3 and 14), basal locomotor activity (LA) (withdrawal day 4), ‘nic-seeking’ behavior (seeking) induced by nic priming (0.4 mg/kg, sc) (withdrawal day 15) and cognition-like deficits in the novel object recognition task (NORT, withdrawal day 17). (**B**) Effects of lor (0.1 mg/kg) on hippocampal neurogenesis during nic withdrawal. Immediately after the last self-administration session, rats were injected with 5-bromo-2’-deoxyuridine (BrdU, 3 × 50 mg/kg, ip) to label proliferating cells. After a 14-day treatment with lor or veh, animals were perfused (Perf), and hippocampal neurogenesis was examined.

Animals (*n* = 42) were trained to press a lever in standard operant chambers (Med-Associates, St. Albans, GA, USA) using a previously published protocol [51] with small modifications. After the training, rats were implanted with an intravenous catheter, as described previously [51]. After recovery, the animals were randomly assigned to either nicotine self-administration (nic; *n* = 30) or a ‘yoked’ control (saline, ysal; *n* = 12) group. Rats were trained to self-administer nicotine (0.03 mg/kg/inf) under an increasing FR (1–5) schedule of reinforcement [68] (21 sessions; maintenance phase; Figure 6A). A detailed procedure of self-administration is described in Supplementary Materials 1.3. 

Immediately after the 20th self-administration session, the anxiolytic-like properties of nicotine were evaluated in the LDB (Figure 6A), as described in Supplementary Materials 1.3.2. and previous publication [69].

#### 4.3.2. Withdrawal

After reaching a stable number of presses on the active lever, animals were subjected to a withdrawal phase during which they were kept in their home cages. In that period, lorcaserin (0.1 or 0.3 mg/kg; nic/lor(0.1): *n* = 10, nic/lor(0.3): *n* = 9) or a vehicle (ysal/veh: *n* = 12, nic/veh: *n* = 11) was administered for 14 days. Lorcaserin doses were chosen based on the FST that was performed in naive animals according to a previously published method [27] and Supplementary Materials 1.2., 2.1., and 2.2. To study the effect of chronic drug administration during nicotine withdrawal, one effective (0.3 mg/kg) and one ineffective (0.1 mg/kg) dose was selected (Figure S1). The following signs of nicotine withdrawal were measured: the anxiety-like behavior in the LDB, depression-like behavior in the FST, locomotor activity, ‘nicotine-seeking’ behavior and cognition-like deficits in the NORT. These fast screening tests were used in the study to minimize the potential confounding due to overlapping procedures.

Effects of Nicotine Withdrawal on Anxiety Behavior in the LDB

To assess the level of anxiety in rats withdrawn from nicotine self-administration, 3–4 h before daily lorcaserin/vehicle injections, the animals were placed in the LDB during early (day 1) and protracted (day 14) abstinence.

Effects of Nicotine Withdrawal on the Behavior in the FST

Following the pretest that was performed on day 2 of nicotine withdrawal, on days 3 and 14 of nicotine withdrawal rats were administered lorcaserin (0.1–0.3 mg/kg) or vehicle, and 60 min later, the behavior of rats was tested for 5 min in the FST. The following parameters were measured: immobility time, swimming and climbing. Design of the FST was based on preliminary data showing that repeated introduction of animals to the FST did not change the measured parameters in the control group.

Effects of Nicotine Withdrawal on Locomotor Activity

Locomotor activity was measured in non-habituated rats during 5- or 30-min trials on day 4 of nicotine withdrawal 60 min after lorcaserin or vehicle administration.

Induction of ‘Nicotine-Seeking’ under Extinction Conditions

On day 15 of nicotine withdrawal, rats were given nicotine (0.4 mg/kg, sc, unconditioned stimulus) and then were immediately reintroduced to the experimental cages to induce ‘drug-seeking’ behavior (i.e., drug-lever presses). During a 2-h session, active-lever presses on the FR5 schedule resulted in an intravenous saline delivery. The strength of ‘nicotine-seeking’ behavior (evidenced by active-lever presses) was compared between animals that received either vehicle or lorcaserin for 14 days.

Effects of Protracted Nicotine Withdrawal on the Behavior in the NORT

Two days after the induction of ‘drug-seeking’ behavior, the rats’ behavior was tested in the NORT. 

For a detailed description of the LDB, FST, locomotor activity, and NORT protocols, see Supplementary Materials (1.2., 1.3.), Figure 6A, and previous publication [70].

### 4.4. Hippocampal Neurogenesis during Nicotine Withdrawal

Separate groups of rats (*n* = 17; ysal: *n* = 9; nic: *n* = 8) were generated in a self-administration procedure (Figure 6B). During nicotine withdrawal, rats were injected with lorcaserin (0.1 mg/kg; ysal/lor(0.1): *n* = 5, nic/lor(0.1): *n* = 4) or its vehicle (ysal/veh: *n* = 4, nic/veh: *n* = 4) for 14 days. Two methods were used to study neurogenesis: immunoenzymatic labeling was used to assess the presence of *K*_i_-67 (a marker of cell proliferation)-, BrdU (a marker of survival of proliferating cells)- and DCX (a marker of immature neurons)-positive cells (*K*_i_-67^+^, BrdU^+^ or DCX^+^, respectively), and triple immunofluorescence labeling (for BrdU, DCX and NeuN) was performed to determine changes in the phenotype of BrdU^+^ cells. Detailed immunostaining protocols are described in Supplementary Materials 1.4. and previous publications [71,72,73].

### 4.5. Statistical Analyses

The data are expressed as the means (±SEM). The sample size calculation was based on our preliminary behavioral (self-administration, FST) and neurobiochemical (hippocampal neurogenesis) data showing that to obtain statistically significant differences between nicotine-withdrawn rats versus control rats a minimum of 8 (behavioral analyses) or 4–5 (neurogenesis analyses) rats per group is necessary. The normality of the data distribution was tested by a Shapiro–Wilk normality test. Grubbs’ method was used to identify outliers that were more than two standard deviations of the mean value and/or significantly (*p* < 0.01) affected normality. The identified outliers (LDB–withdrawal day 1: one rat–nic group; FST–withdrawal day 3: one rat–nic/lor(0.3) group, withdrawal day 14: one rat–nic/lor(0.1) group; ‘nicotine-seeking’: one rat–nic/veh group, one rat–nic/lor(0.1) group) were removed in a given analysis. After examining all the assumptions (i.e., normal distribution, equality of variance), comparisons between means representing changes from the control values were made using a Student’s *t*-test for independent samples (FST/locomotor activity in naive rats; LDB–self-administration session/withdrawal day 1; neurogenesis experiment–cumulative nicotine intake/immunostaining). In the case of the occurrence of unequal variances despite the absence of outliers, Student’s *t*-test with Welch’s correction was used (LDB–self-administration session/withdrawal day 1; immunostaining–% of colocalized BrdU^+^/DCX^+^/total BrdU^+^). In the case of the data in which the data distribution was not normal and did not depend on the presence of outliers, a Mann–Whitney U test was used. A two-way analysis of variance (ANOVA) for repeated measures with interaction was used to analyze the lever presses during the maintenance phase (factors: lever, treatment (nicotine), session). A one-way repeated measures ANOVA was used to analyze the locomotor activity measurements (NORT). Repeated measures ANOVA with interaction was used to process nicotine infusions during the maintenance phase. The number of lever presses during the induction of ‘drug-seeking’ behavior (factors: lever, group/treatment (lorcaserin)), exploration time in the NORT (factors: treatment (lorcaserin), object type/object novelty) and immunostaining data (factors: group (nicotine withdrawal), treatment (lorcaserin)) were analyzed using a two-way ANOVA with interaction. One-way ANOVA was used to analyze the effect of lorcaserin in the FST (in naive rats and during nicotine withdrawal), cumulative nicotine intake, locomotor activity (withdrawal day 4), anxiety-like behavior (withdrawal day 14), and DI in NORT. ANOVA was followed by a post hoc Newman–Keuls test. All comparisons were made with an experiment type I error rate (α) set at *p* < 0.05. Statistics were calculated using GraphPad Prism v.8.3.1 software (GraphPad Software, La Jolla, CA, USA). 

## Data Availability

Data is contained within the article and supplementary material.

## Supplementary Materials

Supplementary Materials can be found at https://www.mdpi.com/1422-0067/22/2/868/s1.

## Abbreviations

5-HT	Serotonin
5-HT_2C_ receptor	Serotonin _2C_ receptor
ANOVA	Analysis of variance
BrdU	5-bromo-2’-deoxyuridine
DA	Dopamine
DCX	Doublecortin
DG	Dentate gyrus
DI	Discrimination index
FR	Fixed ratio
FST	Forced swim test
LDB	Light/dark box test
NORT	Novel object recognition task
