# Supplementary material for: Chronic Lorcaserin Treatment Reverses the Nicotine Withdrawal-Induced Disruptions to Behavior and Maturation in Developing Neurons in the Hippocampus of Rats
Source: Int J Mol Sci. 2021 Jan 16;22(2):868. doi: 10.3390/ijms22020868 (PMC7831001; doi:10.3390/ijms22020868)
Supplement: Supplementary file 1 [file ijms-22-00868-s001.pdf]

1. Supplementary Materials and Methods

1.1. Drugs

BrdU was dissolved in a filtered (Whatman filters, PP w/GMF 0.2 μm; GE Healthcare, Chicago, IL, USA) solution of 0.9% saline and 0.007N NaOH (pH 7.5). Nicotine was dissolved in sterile 0.9% saline. The pH was adjusted to 7.0 using 20% NaOH. In the case of the iv self-administration paradigm, the nicotine solution (0.03 mg/kg/inf) was filtered using Whatman filters. During the induction of 'drug-seeking' behavior, the drug (0.4 mg/kg, sc) was administered at a volume of 1 ml/kg. Lorcaserin (0.1-0.6 mg/kg, sc) was dissolved in sterile 0.9% saline and injected at a volume of 1 ml/kg.

1.2. Effects of Lorcaserin in the FST in Naive Rats

On the first day of the FST, rats were individually placed in a nontransparent cylindrical tank (50-cm high, 23 cm in diameter) filled with water (30-cm deep, 25 ± 1°C), and they remained there for 15 min (the pretest). The rats were then removed, dried and returned to their home cages. On day 2, the rats were treated with lorcaserin (0.1-0.6 mg/kg, n = 8/group) or its vehicle (n = 8), and 60 min later, the behavior of the rats was tested for 5 min in the FST under identical conditions. The following parameters were measured by two experimenters: immobility time, swimming and climbing. All the test sessions were recorded by a video camera to allow repeating the doubtful measurements.

To verify the specificity of the effects seen in the FST, the effect of the active dose of lorcaserin (0.3 mg/kg; in the FST) on locomotor activity was tested in non-habituated (spontaneous locomotor activity) rats (veh: n = 8; lor(0.3): n = 8). Locomotor activity was recorded individually for each animal in Opto-Varimex cages (Columbus Instruments, USA). Interruptions of the photobeams resulted in the measurement of horizontal locomotor activity, which was defined as the distance traveled (expressed in cm). Measurements of locomotor activity were recorded during 5- or 30-min trials 60 min after injection with lorcaserin (0.3 mg/kg) or its vehicle.

1.3. Behavioral Effects of Nicotine Intake and its Withdrawal

1.3.1. Training and Intravenous Catheter Implantation

After 16-18-h water deprivation, animals were trained for 5 days to press a lever for 2 h in standard operant chambers (Med-Associates, St. Albans, GA, USA) under a fixed ratio (FR) 1 schedule of water reinforcement. Two days after lever press training, during which animals had free access to food and water, rats were anesthetized with a solution containing ketamine (20 mg/kg, im; Biowet, Puławy, Poland) and dexmedetomidine (0.1 mg/kg, im; Orion Corporation, Espoo, Finland), and then they were implanted with a silastic catheter in the external right jugular vein. Following catheter implantation, all animals recovered for 7-10 days. For two days after the surgery, animals received 4-5 ml of 0.9% NaCl/5% glucose solution (sc), and for three days, they were given the anti-inflammatory/analgesic drug meloxicam (0.04 mg/kg, sc; Metacam, Boehringer Ingelheim, Ingelheim/Rhein, Germany). Catheters were flushed daily with 0.2 ml of a sterile 0.9% saline solution containing cephazolin (100 mg/ml; Polpharma, Warszawa, Poland) and heparin (100 IU/ml; Polfa, Warszawa, Poland) to maintain catheter function.

1.3.2. Maintenance of Nicotine Self-Administration

Rats were given access to nicotine during the daily 2-h session (6 days/week). The ambient light was on throughout each session. Each press on the active lever (i.e., the number of presses was gradually increased from FR1 to FR5) resulted in an infusion of nicotine (0.03 mg/kg per 0.1 ml) and a 5-second presentation of a conditioned stimulus (illumination of a stimulus light + tone). Following each infusion, there was a 20-s time-out period during which responding was recorded but not

reinforced. Rats were tested simultaneously in two groups with one of the rat group serving as the 'yoked' control that received an injection of saline (ysal) each time a response-contingent injection of nicotine was self-administered by the paired rat from the second group (nic). Saline passive injections were accompanied by the presentation of cues (light + tone). Acquisition of the conditioned operant response lasted a minimum of 21 days until the subjects achieved the criteria for stable responding: stable self-administration over the last 3 sessions with a standard deviation within those days that was <10% of the average.

Effects of Nicotine in the LDB

The animals were kept in total darkness for 30 min before the test and throughout the whole experiment. Following habituation, the animals were placed for 10 min in LDB cages (TSE Systems, Bad Homburg, Germany). Each arena, 45 cm × 45 cm × 45 cm, consisted of two compartments: one dark compartment (made of black acrylic, covering 1/3 of the total cage area) and one light compartment (light intensity - 60 lx, made of transparent acrylic, covering the remaining 2/3 of the cage), separated from each other by a wall equipped with a central tunnel gate (height x width: 11 cm x 8 cm). The experimental cages were located in soundproof cabinets containing integrated infrared sensors that detected the animals along the X-, Y- (horizontal level) and Z- (vertical level) axes. The sensor frames on the X-, Y- and Z-axes were equipped with 32 sensor pairs mounted 14 mm from each other. The cages were equipped with fans providing background noise (65 dB). The behavior of animals (locomotor activity, the amount of time spent in each compartment, number of transitions between the compartments) was automatically recorded by a camera mounted on the ceiling of the cabinet and was analyzed using Fear Conditioning software (TSE Systems).

1.3.3. Nicotine Withdrawal

Effects of Protracted Nicotine Withdrawal on the Behavior in the NORT

24 h before testing, the rats were habituated to the arena (without any objects) for 5 min. The rats were tested in a dimly lit (25 lx) open field made of dull gray plastic (length × width × height: 66 cm × 56 cm × 30 cm). After each measurement, the floor was cleaned and dried. The following day, the test comprised two 3-min trials separated by an intertrial interval of 1 h. During the first trial (familiarization, T1), two identical objects (A1 and A2) were presented in opposite corners, and they were approximately 10 cm from the walls of the open field. In the second trial (retention, T2), one of the objects was replaced with a novel object (A = familiar and B = novel). The animals were returned to the home cage after T1. The objects used in the test included a glass bulb filled with gravel and a plastic bottle filled with sand. The heights of the objects were comparable (ca. 12 cm), and both objects were heavy enough to not be displaced by the animals. Half of the animals from each group identified the glass bulb as a novel object, and the other half identified the plastic bottle. The location of the novel object in the recognition trial was randomly assigned for each rat. The exploration of an object was defined by looking, licking, sniffing or touching the object while sniffing but not by leaning against, standing or sitting on the object. Any rat spending less than 5 s exploring the two objects within 3 min of T1 or T2 was eliminated from the study. The behavior of the rats was recorded using a camera placed above the arena and connected to an Any-maze® tracking system (Stoelting Co., Wood Dale, IL, USA). An experimenter blinded to the treatment conditions manually assessed the exploration time. Additionally, the distance traveled was automatically measured using an Any-maze® tracking system. Based on the exploration time (E) of the two objects, the discrimination index (DI) was calculated as $DI=(EB - EA)/(EA + EB)$.

1.4. The Hippocampal Neurogenesis during Nicotine Withdrawal

1.4.1. Immunohistochemistry

Immediately after the 21st self-administration session, rats were given three injections of BrdU (50 mg/kg, ip) at 6-h intervals during early withdrawal. During the withdrawal period, animals were kept in their home cages and received lorcaserin (0.1 mg/kg) or its vehicle once daily. At 14 days post-cessation, animals were deeply anesthetized with a sublethal dose of sodium pentobarbital (90 mg/kg) and pentobarbital (18 mg/kg) (ip; Morbital, PGF Cefarm, Kraków, Poland) and were transcardially perfused with 0.9% NaCl, which was followed by buffered 4% paraformaldehyde (VWR International, Radnor, PA, USA). After perfusion, the brains were isolated from the skull and postfixed in a buffered solution of 4% paraformaldehyde for 24 h at 4 °C. After the postfixation period, the brains were cut into 50 μm thick sections (covering the entire hippocampus) using a Leica VT-1000S vibratome (Leica Microsystems, Heidelberg, Germany).

Single Staining for BrdU

Every ninth section throughout the entire hippocampus was processed for BrdU immunohistochemistry (8 sections for each subject). Free-floating sections were first denatured in a solution containing 50% formamide/2xSSC (saline-sodium citrate buffer, pH 7.0; Sigma-Aldrich, St. Louis, MO, USA) for 2 h at 65 °C. Next, the sections were washed twice in 2xSSC buffer for 5 min, and then they were incubated in 2M HCl for 30 min at 37 °C, before being incubated in 0.1M borate buffer (0.1M boric acid + NaOH; pH 8.5) for 10 min at room temperature. Subsequently, the brain sections were rinsed with 0.01M PBS and then were incubated in blocking buffer (5% normal goat serum; Vector Laboratories, Peterborough, UK and 0.3% Triton X-100 in 0.01M PBS) for 1 h. Finally, the sections were incubated (48 h at 4 °C) with primary monoclonal anti-BrdU mouse antibodies (1:200; Roche Diagnostics, Indianapolis, IN, USA; cat no. 11170376001) in 3% normal goat serum with 0.3% Triton X-100 in 0.01M PBS. Reactions were visualized with biotinylated goat anti-mouse IgG (1:200, 1 h; Vector Laboratories), which was followed by incubation with avidin-biotin-horseradish peroxidase complex (1:200, 1 h; VECTASTAIN Elite ABC HRP Kit, Vector Laboratories), 3,3′-diaminobenzidine tetrahydrochloride (DAB)-nickel solution (0.02% DAB + 0.03% $NiCl_2$ in 0.01M PBS) and 0.01% H_2O_2, which resulted in a dark gray color of immunoreactive cells.

Single Staining for K_i-67 or DCX

Free-floating brain sections were rinsed and incubated for 1 h in a blocking buffer (5% normal goat serum for K_i-67 or 5% normal rabbit serum for DCX labeling; Vector Laboratories and 0.3% Triton X-100 in 0.01M PBS). Next, sections were incubated (48 h at 4 °C) with a primary antibody: rabbit polyclonal anti-K_i-67 (1:750; Abcam, Cambridge, UK; cat no. ab15580) or goat polyclonal anti-DCX (1:500; Santa Cruz Biotechnology, Santa Cruz, CA, USA; cat no. sc-8066), diluted in appropriate 3% normal serum with 0.3% Triton X-100 in 0.01M PBS. The reaction was visualized using a biotinylated goat anti-rabbit (1:200; Vector Laboratories) or rabbit anti-goat (1:200; Vector Laboratories) IgG, which was followed by incubation with a peroxidase complex (1:200, 1 h; Vectastain Elite ABC Kit), 0.02% DAB solution, and 0.01% H_2O_2, and the result was a brown color in cells that were immunopositive for DCX. A DAB-nickel solution (0.02% DAB+0.03% $NiCl_2$ in 0.01M PBS) with 0.01% H_2O_2 was used for K_i-67.

1.4.2. Immunofluorescence

Two sections containing the proximal and distal parts of the hippocampus were selected for triple-label immunofluorescence. The sections were processed for DNA denaturation (as described above) and then were blocked in blocking buffer (5% normal donkey serum; Jackson Immunoresearch Laboratories, West Grove, PA, USA and 0.3% Triton X-100 in 0.01M PBS) for 1 h. Next, the sections were incubated (48 h at 4 °C) with a cocktail of BrdU (monoclonal rat 1:300; Accurate Chemical and Scientific Corp., Westbury, NY, USA; cat no. AB6326)-, DCX (polyclonal goat 1:500; Santa Cruz Biotechnology)- and NeuN (monoclonal mouse 1:1000; EMD Millipore, Temecula, CA, USA; cat no.

MAB377)-specific primary antibodies in 3% normal donkey serum with 0.3% Triton X-100 in 0.01M PBS. After washing in 0.01M PBS, the sections were incubated (overnight at 4 °C) in a mixture of secondary antibodies (Alexa 488-conjugated donkey anti-rat IgG 1:200; Thermo Fisher Scientific, Rockford, IL, USA, Cy3-conjugated donkey anti-goat IgG 1:300; Jackson Immunoresearch Laboratories and Cy5-conjugated donkey anti-mouse IgG 1:300; Jackson Immunoresearch Laboratories) in 3% normal donkey serum with 0.3% Triton X-100 in 0.01M PBS (colocalization of BrdU, DCX and/or NeuN immunoreactivities). Finally, the sections were rinsed in 0.01M PBS, mounted onto gelatin-coated slides and coverslipped with medium containing 0.01M PBS-buffered glycerol.

1.4.3. Quantitative Evaluation of Staining

For immunoenzymatic staining, the number of immunoreactive ($BrdU^+$, K_i-67^+ or DCX^+) cells in the dentate gyrus (DG) of the hippocampus was estimated under a light microscope (Leica, DM 6000B; Leica Microsystems) equipped with a motorized stage (Ludl Electronic Products, Hawthorne, NY, USA). Briefly, every ninth section along the rostrocaudal axis of the hippocampal formation was analyzed with a 63× planapochromat lens using Stereo Investigator software v.8.0 (MBF Bioscience, Williston, VT, USA). Cells appearing in the upper focal plane were omitted to prevent counting the tops of cells (−5 μm of the topmost surface of the section). For each animal, the mean numerical density of immunoreactive cells was calculated from the sum of the counts made within the optical dissectors. The final results were presented as the number of immunoreactive cells in the DG of the hippocampus, which was calculated automatically by Stereo Investigator software per 1 mm^3 hippocampal area.

For triple-label immunofluorescence, two sections per subject across the studied region were analyzed using a confocal microscope (Leica TCS SP8, WLL, Leica Microsystems) with excitation wavelengths of 499 nm (Alexa 488), 548 nm (Cy3) and 649 nm (Cy5). The sections were scanned using a 63x objective (HC PL APO CS2 63x/1.40 OIL) along the *Z*-axis (Z-step size: 1-μm, scan speed: 400 Hz, frame size: 1024 x 1024). *Z*-plane stacks of images were collected at every location within the hippocampal DG in which $BrdU^+$ cells were visible. Images were further examined using Leica Application Suite X v.3.5.2.18963 (LAS X; Leica Microsystems) software to observe the phenotype of $BrdU^+$ cells. For each rat, the number of single ($BrdU^+$)-, double ($BrdU^+/DCX^+$ or $BrdU^+/NeuN^+$)- and triple ($BrdU^+/DCX^+/NeuN^+$)-labeled cells was estimated. The results are presented as the mean number of cells per section and the percent of colocalized individual cell type per total BrdU cell population per region.

1.4.4. Preparation of Photomicrographs for Data Presentation

To present examples of cells that exhibited immunoreactivity for the neurogenesis markers used in the hippocampus, immunoenzymatically stained sections were imaged with an Aperio ScanScope slide scanner (Aperio UK, Oxford, UK), while immunofluorescent staining was presented by using a representative *Z*-plane stack of images from a control ysal animal. Final photomicrographs were compiled with ImageJ (NIH, Bethesda, MA, USA) and CorelDraw v.11.0 (Corel Corporation, Ottawa, Canada) software.

2. Supporting Results

2.1. Lorcaserin Exerts Antidepressant Properties in the FST in Naive Rats

Pretreatment of rats with lorcaserin (0.1-0.6 mg/kg) affected the immobility time ($F(3,28) = 5.92$, $p = 0.0029$). The post hoc Newman-Keuls test showed that lorcaserin at a higher dose (0.6 mg/kg) significantly increased (by 40%; $p = 0.039$) the immobility time of rats, while its lower dose (0.3 mg/kg) nonsignificantly reduced (by 23%; $p = 0.15$) the time (Figure S1). A single dose comparison with the control group revealed that the 0.3-mg/kg dose reduced immobility time compared to that of the vehicle-treated rats (t = 2.42, df = 14, $p = 0.03$) (Figure S1). Pretreatment with lorcaserin altered swimming behavior ($F(3,28) = 6.30$, $p = 0.0021$). Post hoc analysis showed that lorcaserin at a dose of 0.3 mg/kg increased (by 44%; $p = 0.03$) the swimming time in rats (Figure S1). There was no change in climbing behavior in rats pretreated with lorcaserin (0.1-0.6 mg/kg) (F(3,28) = 0.41, $p = 0.74$) (Figure S1). Overall, the 0.3-mg/kg dose of lorcaserin exhibited an antidepressant-like effect (i.e., shortened the immobility time and increased swimming behavior), while the 0.6-mg/kg dose induced depression-like behavior (i.e., increased immobility time) in naive rats.

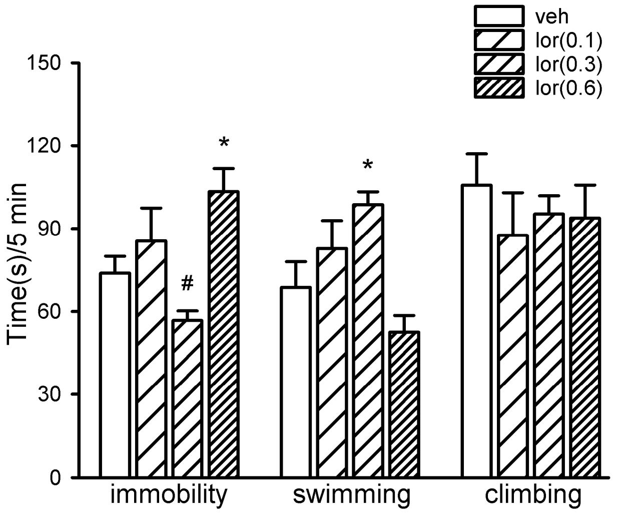

Figure S1: Effects of lorcaserin on the behavior of naive rats in the forced swim test (FST). The data are expressed as the means (± SEM). veh: $n = 8$; lor(0.1): $n = 8$; lor(0.3): $n = 8$; lor(0.6): $n = 8$. $^{*}p < 0.05$ versus veh; $^{\#}p < 0.05$ versus veh. Veh, vehicle, lor, lorcaserin.

2.2. Active Dose of Lorcaserin in the FST Does Not Affect Locomotor Activity of the Animals

Administration of lorcaserin (0.3 mg/kg; active dose in the FST) did not alter the locomotor activity of the animals either during a 5 (distance traveled: veh: 947.63 ± 18.34 cm, lor(0.3): 844.89 ± 55.49 cm; t = 1.76, df = 8.51, $p = 0.11$)- or 30 (distance traveled: veh: 2208.80 ± 149.92 cm, lor(0.3): 2232.16 ± 232.17 cm; t = 0.085, df = 14, $p = 0.93$)-min measurement period, indicating that the effect of lorcaserin (0.3 mg/kg) in the FST was specific.

2.3. Cumulative Nicotine Intake in Rats within 21 Self-Administration Sessions

Total nicotine intake during 21 sessions did not differ between the three assigned groups that received vehicle or lorcaserin (0.1 or 0.3 mg/kg) during a 14-day withdrawal period ($F(2,27) = 0.48$, $p = 0.63$) (Figure S2).

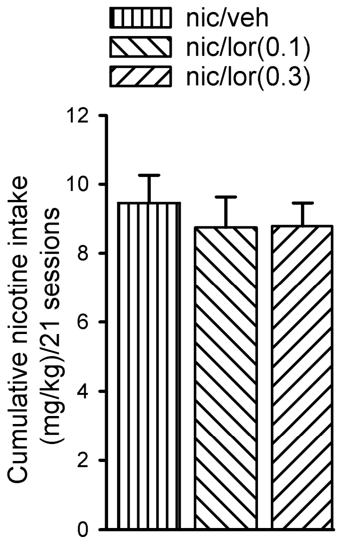

Figure S2: Cumulative nicotine (0.03 mg/kg/inf; nic) intake in rats within 21 self-administration sessions. The data are expressed as the means (± SEM). nic/veh: *n* = 11; nic/lor(0.1): *n* = 10; nic/lor(0.3): *n* = 9. Veh, vehicle, lor, lorcaserin.

2.4. The Effects of Chronic Lorcaserin Administration on the Behavior of Nicotine-Withdrawn Rats in the NORT

The time spent exploring two identical objects (Trial 1) was not changed following lorcaserin treatment (interaction: $F(3,76) = 1.04$, $p = 0.38$) (Figure S3A). No significant treatment effect was observed in the distance traveled by rats in the familiarization and retention trials (interaction: $F(3,38) = 0.56$, $p = 0.65$) (Figure S3B).

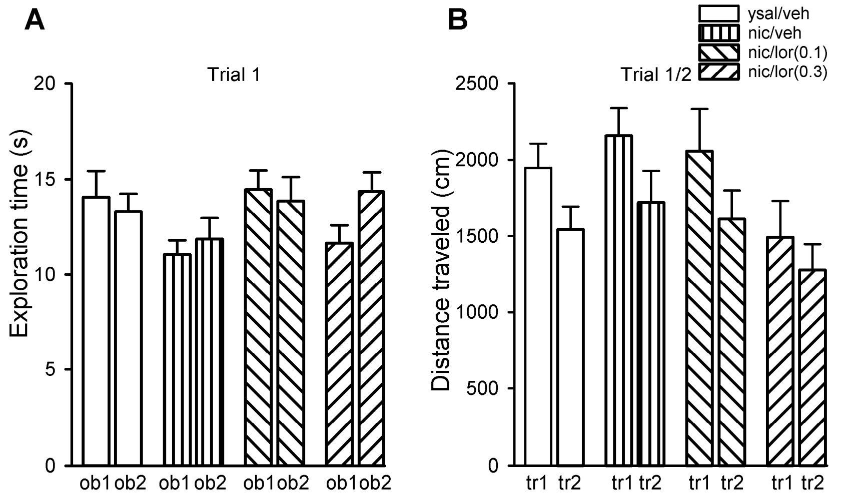

Figure S3: Effects of chronic lorcaserin administration on the behavioral parameters in the novel object recognition task (NORT) in nicotine-withdrawn rats. (**A**) Effects of chronic lorcaserin (0.1-0.3 mg/kg, lor(0.1/0.3)) treatment during nicotine (nic) withdrawal on the time spent exploring two identical objects on withdrawal day 17. (**B**) Effects of lor (0.1/0.3) on the distance traveled in the NORT in nic-withdrawn rats. The data are expressed as the means (± SEM). ysal/veh: *n* = 12; nic/veh: *n* = 11; nic/lor(0.1): *n* = 10; nic/lor(0.3): *n* = 9.

2.5. Rats Used in the Biochemical Analyses Self-Administer Nicotine at Levels that are Comparable to the Animals Tested in Behavioral Assays

Within 21 sessions, nicotine changed the number of lever presses in rats (interaction: $F(20,600) = 9.67$, $p < 0.001$). Post hoc Newman–Keuls analysis revealed that the number of active-lever presses in rats that self-administered nicotine was significantly higher than the number of inactive-lever presses in that group (sessions: 9, 15; $p < 0.01$, sessions: 10–14, 16–21; $p < 0.001$), and the number was also higher than that of the active-lever presses in the ysal group (session: 8; $p < 0.01$, sessions: 9-21; $p < 0.001$) (Figure S4A). The number of nicotine infusions within 21 sessions was altered in rats ($F(20,140) = 2.85$, $p < 0.001$). Post hoc Newman–Keuls analysis showed that the number of infusions during session 3 was significantly lower than it was during session 1 ($p < 0.01$) (Figure S4B). Total nicotine intake during 21 sessions did not differ between the two assigned groups that received vehicle or lorcaserin (0.1 mg/kg) during a 14-day withdrawal period (t = 0.00, df = 6, $p > 0.05$) (Figure S4C).

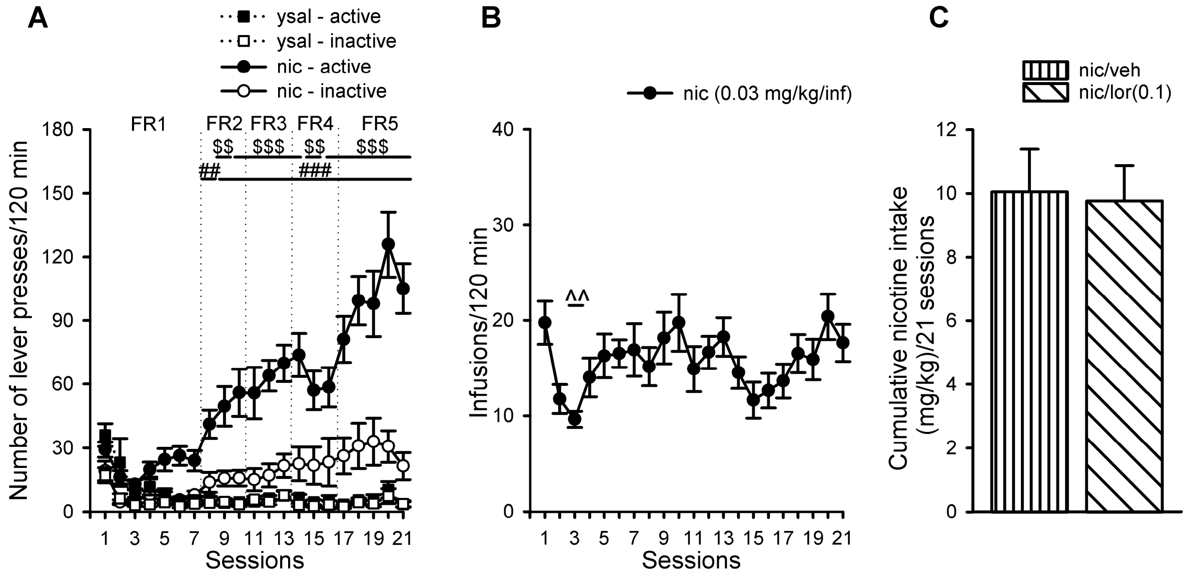

Figure S4: Nicotine self-administration in rats. (**A**) The number of lever presses in rats self-administering nicotine (0.03 mg/kg/inf; nic) and rats that received saline (ysal) under an increasing schedule of reinforcement (FR1-5). (**B**) Nic infusions throughout 21 self-administration sessions. (**C**) Cumulative nic intake in rats assigned to two groups (receiving vehicle (veh) or lorcaserin (0.1 mg/kg; lor(0.1)) during drug withdrawal. The data are expressed as the means (± SEM). (**A**) ysal: $n = 9$; nic: $n = 8$, (**B**) nic: $n = 8$, (**C**) nic/veh: $n = 4$; nic/lor(0.1): $n = 4$. (**A**) $^{\$\$}p < 0.01$, $^{\$\$\$}p < 0.001$ versus nic-inactive; $^{\#\#}p < 0.01$, $^{\#\#\#}p < 0.001$ versus ysal-active. (**B**) $^{\wedge\wedge}p < 0.01$ versus session 1.
